# How to improve vital sign data quality for use in clinical decision support systems? A qualitative study in nine Swedish emergency departments

**DOI:** 10.1186/s12911-016-0305-4

**Published:** 2016-06-04

**Authors:** Niclas Skyttberg, Joana Vicente, Rong Chen, Hans Blomqvist, Sabine Koch

**Affiliations:** Capio St Görans Hospital, 112 81 Stockholm, Sweden; Health Informatics Centre, Department of Learning, Informatics, Management, and Ethics, Karolinska Institutet, 171 77 Stockholm, Sweden; Cambio Healthcare Systems, Stockholm, Sweden; Department of Anaesthesia and Intensive Care, Karolinska University Hospital, 171 76 Stockholm, Sweden

**Keywords:** Computer-assisted decision making, Data quality, Electronic health records, Emergency care, Medical informatics, Vital signs

## Abstract

**Background:**

Vital sign data are important for clinical decision making in emergency care. Clinical Decision Support Systems (CDSS) have been advocated to increase patient safety and quality of care. However, the efficiency of CDSS depends on the quality of the underlying vital sign data. Therefore, possible factors affecting vital sign data quality need to be understood.

This study aims to explore the factors affecting vital sign data quality in Swedish emergency departments and to determine in how far clinicians perceive vital sign data to be fit for use in clinical decision support systems. A further aim of the study is to provide recommendations on how to improve vital sign data quality in emergency departments.

**Methods:**

Semi-structured interviews were conducted with sixteen physicians and nurses from nine hospitals and vital sign documentation templates were collected and analysed. Follow-up interviews and process observations were done at three of the hospitals to verify the results. Content analysis with constant comparison of the data was used to analyse and categorize the collected data.

**Results:**

Factors related to care process and information technology were perceived to affect vital sign data quality. Despite electronic health records (EHRs) being available in all hospitals, these were not always used for vital sign documentation. Only four out of nine sites had a completely digitalized vital sign documentation flow and paper-based triage records were perceived to provide a better mobile workflow support than EHRs. Observed documentation practices resulted in low currency, completeness, and interoperability of the vital signs. To improve vital sign data quality, we propose to standardize the care process, improve the digital documentation support, provide workflow support, ensure interoperability and perform quality control.

**Conclusions:**

Vital sign data quality in Swedish emergency departments is currently not fit for use by CDSS. To address both technical and organisational challenges, we propose five steps for vital sign data quality improvement to be implemented in emergency care settings.

**Electronic supplementary material:**

The online version of this article (doi:10.1186/s12911-016-0305-4) contains supplementary material, which is available to authorized users.

## Background

Vital sign data are important in emergency care decision making, especially for prioritization and identification of severe illness. To screen for sepsis, vital signs are essential in early detection [1, 2], and it is well known that rapid detection and early treatment are key factors to improve patient survival [3]. Studies in the emergency department show that vital signs can be used in predicting cardiac arrest [4, 5] and sepsis outcome [6]. Further, the vital signs are broadly used in clinical care in the calculation of warning scores that are aiming at predicting clinical deterioration [7, 8]. In emergency care, the first use of the vital signs is often in the triage of arriving patients [9–11]. Reports show that triage systems are used in almost all Swedish emergency departments [12, 13] and that the majority of the hospitals use the rapid emergency triage and treatment system (RETTS) [10]. The common denominator of most triage models is that they use vital sign measurements to calculate a triage score.

Clinical Decision Support Systems (CDSS) using data from electronic health records (EHR) are advocated to improve clinical outcome [14, 15], quality and patient safety [16]. In emergency care, some studies have indicated that triage CDSS may provide reliable calculations of triage severity [17] and even improve risk assessment [18], while others have questioned the readiness for automation [19] . To be trustworthy the CDSS need to provide reliable advice to the clinician. The quality of the CDSS recommendations will depend on the quality of the underlying data in the EHR [20]. When comparing to traditional definitions of data quality, stating that quality is good enough when the data can fulfil the intended purpose [21–23], this means that the underlying data has to be able to support reliable calculations in the CDSS. To provide reliable triage scores, the vital sign data needs to be correct, complete, and timely available. A comprehensive review by Weiskopf and Weng describes these three main data quality categories as; Correctness, Completeness, and Currency [24], where the correctness of the data indicates to how true the documented vital signs are, completeness refers to whether all expected vital signs are actually registered in the EHR and currency is linked to the temporal aspects of the documented vital signs.

The yearly report on healthcare IT and e-health in Sweden [25] shows that EHRs have a penetration of a 100 % in Swedish emergency care hospitals, and based on this high penetration combined with the wide use of triage systems, a high completeness of vital signs may be expected. However, some studies indicate problems with vital sign data quality. A study by di Martino et al. show that the completeness of the vital signs needs improvement [26], further Genes et al. [27] state that vital sign data correctness is relatively low, and finally Ward et al. [28] question the operational integrity of the time stamps after EHR implementation. Less is known about which factors affect emergency care EHR data quality and in what way they affect it [26, 29]. The di Martino [26] study shows that a clinical improvement program may increase completeness and Wager et al. [29] state that the entry device will have an effect on correctness and currency. If CDSS is expected to have an impact on patient safety and quality, more knowledge is needed on how to assure and improve clinical data quality. This knowledge can be used both to support CDSS development and in clinical quality improvement work.

### Objective

The aim of this study is three-fold. Firstly, to explore the factors affecting vital sign data quality during measurement and documentation in Swedish emergency care. Secondly, to determine how far clinicians perceive documented vital sign data to be fit for use in clinical decision support systems. Thirdly, to provide recommendations on how to improve vital sign data quality in emergency care.

## Methods

**Fig. 1:**
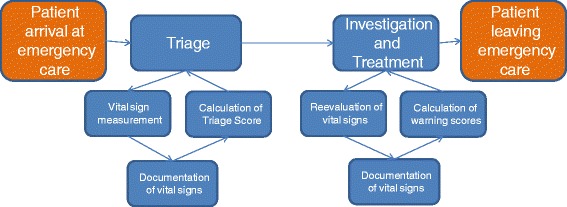
Emergency care process. Vital sign measurement and documentation in the emergency care process

We explored the factors affecting vital sign data quality in emergency care using a qualitative approach. The explored process of vital sign collection and use is described in Fig. 1. Data collection was done through semi-structured interviews, observations and analysis of documentation templates in nine purposefully selected Swedish emergency departments. We used content analysis with constant comparison to categorize the data [30–32].

The study was performed during a period of ten months (August 2014 – May 2015).

### Setting and participants

Inclusion criteria for the participants were a degree as Medical Doctor (MD) or Registered Nurse (RN) with a minimum of five years of experience in emergency care, in particular, triage and vital sign documentation. Quality managers at the sites were contacted and helped to identify a total of sixteen participants that fulfilled the inclusion criteria. The participants were invited by email and all accepted to participate in the study.

Sites were included aiming at variability in size and regional distribution. In total, nine hospitals (Table 1) were included in the study, five university hospitals (UH) and four secondary referral centres (SRC). Three different Electronic Health Record Systems were used at these sites.

**Table 1:** An overview of the performed interviews

SiteNumber	Type	ED Visits	#MDs Interviewed	#RNs Interviewed
Site 1	UH	65000	1	1
Site 2	SRC	40000		3
Site 3	SRC	30000	1	
Site 4	UH	97000		2
Site 5	UH	53000		1
Site 6	SRC	80000		1
Site 7	SRC	39000		3
Site 8	UH	45000		2
Site 9	UH	53000		1

### Data collection

Data was collected in three subsequent steps. First, sixteen semi-structured interviews were conducted at nine different sites (Table 1). The interviews were performed using a semi-structured interview guide (included in the Additional file 1) covering different aspects of data quality such as completeness, correctness, and currency. The interview guide also aimed to cover how the participants experienced existing vital sign data quality and perceived opportunities to increase vital sign data quality in the EHRs. The guide was tested in a pilot interview. All interviews were voice recorded, performed in Swedish, either on-site or by telephone, and lasted for about 30 min.

Confirmation was sought with the participants to verify the findings from the interviews. This was done through second round interviews with open-ended discussions on the themes and categories found in the results. The participants were selected as they represented different documentation practices in the initial interviews. The interviews were performed on site in Swedish, lasted for about 45 min each and were voice recorded.

After the interviews, observations were performed at three selected sites aiming to complement the data collected during the interviews. Sites No. 2, 4 and 8 were purposefully selected for the observations as they represented examples of three different documentation practices that were found in the initial interviews (Table 3). Observations focused on vital sign measurements and documentation in the emergency departments, aiming to cover a variation in locations, staff, and clinical situations. The observations aimed to confirm the findings from the interviews in clinical practice and they lasted about 60 min per site. The observations were performed by the researchers together with a nurse from the site. The observers were free to move around the emergency department during the observations and during the observations photos and field notes were taken and samples of documentation templates were collected from the emergency departments. From the observations, a structured report was written. The observation report protocol is included in the Additional file 1.

Two researchers (NS and JV) performed the interviews and observations. A third and fourth researcher (SK and RC) continuously gave feedback on interviews and results.

### Data analysis

The recorded interviews were transcribed and coding was done by reading through the transcripts. Quotes and meaning units were translated into English. The codes were inductively abstracted into categories and themes.

Transcription, coding, abstraction and rechecks with the audio recordings were done continuously. Two researchers (NS and JV) performed coding and abstraction separately. Discussions in the research group were held to compare coding and emerging categories, making concepts and categories change and evolve during the process. Changes in the coding and categories diminished over time and eventually a consensus on categories was reached in the research group. Interviews were performed until no new concepts or categories emerged. After consensus and saturation, the results from the analysis were confirmed by three of the initial participants in the form of open second-round interviews. These interviews were transcribed and coded in the same way as the first-round interviews. Additional data such as documentation templates and field notes from the observations were subsequently included in the analysis. This further confirmed saturation and strengthened our confidence in the interpretation of the initial interviews.

### Ethical considerations

Ethical approval was applied for at the Stockholm Ethical Committee but not considered to be needed (Dnr 2014/1207-31/4). Information on the study was given in advance and informed consent was obtained. Participation was voluntary and confidentiality was assured. To assure confidentiality none of the quotes are connected to the sites in the publication.

## Results

### Factors affecting vital sign data quality

The factors resulting from the content analysis are presented according to the main themes and categories that were found in our analysis (Table 2). The table includes examples of subcategories, meaning units and corresponding quotes. The quotes are related to the type of documentation practice found at the site; paper-based documentation, mixed documentation, and digital documentation (Table 3).

**Table 2:** Factors affecting vital sign data quality

Themes, categories and example quotes				
Theme	Main category	Subcategory	Meaning unit (examples)	Quote and type of documentation practice
Care process	Standardized process	Standardized triage	Standardized Triage - Securing Vital Sign measurements	“We do triage on all patients arriving at the emergency department. No difference if they are arriving by ambulance or walking in. A short history and vital sign measurements are included in all patients.” PD
		Standardized documentation	Standard of documentation improves completeness	“I think it has improved a lot (data quality of vital sign). Before the structured workflow was set, respiratory rate was not completed as often as today." DD
		Failure to comply	Failure to comply - Individual Clinical Judgement	“If a patient has a minor complaint the standard may not be experienced as relevant. In those cases, there may be failure to comply” DD
		Lack of standard	Lack of Standard in repeated measurement documentation	“A patient was kept close to the nurse desk with automated continuous vital sign measurements for hours. Only two recordings were entered into the EHR.” DD
	Management	Quality control	Government Control of Care Quality	“We received feedback from the National Board of Health and Welfare considering our documentation of vital signs. That has made us change routines on documentation and the way we follow up on compliance with documentation standards” DD
		Change management	Resistance to change - switching to digitalized flow	[switch to digitalized flow] “It wasn´t completely easy to achieve. At first, the physicians lacked the paper. But nowadays no one wants to switch back.” DD
		Education/training of staff	Understanding of documentation importance	“You have to educate to increase the understanding why it [documentation] is important. Otherwise, there may be neglect of registrations.” MD
	Competence and knowledge	Method and equipment	Error sources - temperature, ear wax	“When it comes to temperature measurements there may be problems due to simple error sources, like wax in the ear canal.” MD
		Clinical competence	Clinical Validity check	“You cannot always trust the device. You have to make a clinical validity check. If there is a problem, you may have to recheck or change method.” DD
Information technology	Workflow support	Mobility	Mobile documentation required when switching to digitalized flow	“Unless we get access to computers at every room or more mobile ways of working, like iPADs we will likely hold on to the paper triage record” PD
		Overviews	Overview of vital sign measurements	“We need a good overview of measurements so that they can be followed over time.” PD
		Checklists	Process overview and checklist.	“What we lack in the EHR is a usable alternative to paper-based triage record. It should provide overviews and checklists to make sure that everything that should be done gets done and that nothing is forgotten” PD
		Calculation support	Automatic calculation of triage score	“We enter the short history and vital signs in the EHR and with a click, the triage colour will be calculated.” DD
	Documentation support	Structured documentation	Documentation templates - anxiety about forgetting	“It makes sure that everything gets done and that we all do it the same way. It will decrease anxiety about forgetting. “PD
		Logical controls	Logical controls - dictation and free text	“We use dictation to enter the vital signs into the EHR. It will be entered in free text. There are no built-in logical controls.” MD
		Completeness checks	Completeness checks	“To complete the triage all vital signs have to be registered. It is a part of the triage process and the system demands a full set.” DD
		Automatic registrations	Automatic registrations of measurements to improve completeness	“Automatic registration of repeated measurements would really improve documentation. If patients are measured every 15 min, there is no time to manually register all measurements.” DD
	Interoperability	Interoperability within system	Reuse of information between modules in EHR	“We are working in our acute care module. We don’t want to use separate parts of the system making double entries" DD
		Interoperability between system	Sharing information with pre-hospital records	“Vital signs will be measured in the ambulance. We will manually copy them into our EHR." DD

Footnote: In the table the following abbreviations are used in relation to the quotes; Paper-based documentation (PD), Mixed documentation (MD) and Digital documentation (DD)

**Table 3:** Documentation practice

Documentation practice	Description	Number of sites
Paper-based documentation	Documentation on a structured paper-based template and later scanned into the EHR in pdf format. No entries of vital signs were done in the EHR.	2 Sites
Mixed Documentation	Done on a paper-based template and later transferred into a digital EHR template.	3 sites
Digital documentation	Documentation on a digital template	4 sites

#### Care process

##### A standardized process

The interviews showed a perceived importance in following a predefined workflow to increase the quality of vital sign data measurement and documentation. Without a standardized process, individual staff considerations guided decisions about when and how to measure and document vital signs. Hence, a standardized process was perceived to increase quality by reducing individual variations.

The results showed that most patients were expected to be triaged early after arrival at the emergency department. “*We do triage all patients arriving at the emergency department. No difference if they are arriving by ambulance or walking in. A short history and measurement of the vital signs are expected in all patients*.” (digital documentation). All but one site used the rapid emergency triage and treatment system (RETTS) as the triage system and usually, triage was expected to be completed within 15 min of arrival. However, at two of the sites triage was only performed if there was a waiting time to see the doctor. If there was no waiting time, vital signs were measured at the physicians’ initiative or order only.

Switching to a standardised workflow, where all patients were expected to be triaged, was mentioned to improve the measurements and documentation of vital signs. As discussed on the example of respiratory rate in one interview; “*I think it has improved a lot (data quality of vital sign). Before the structured workflow was set, respiratory rate was not completed as often as today*” (digital documentation).

Repeated measurements of vital signs were mentioned as an area with poor data quality. This was attributed to the lack of a standardized process “*There are difficulties in finding a routine both in measurement and documentation (in the re-evaluation of vital signs*)” (digital documentation). One interpretation of the difference between triage and repeated measurements is that the start of the emergency care process is easier to standardize. The later part of the process may be more diverging and depending on the patient's complaints (see also Fig. 1). One of the sites mentioned having implemented vital sign rounds at the emergency department. Patients with high triage scores were expected to have their vital signs rechecked every 15 to 30 min. The aim was to avoid unnoticed patient deterioration by standardizing reevaluation of the vital signs.

Even with a set standard in place not all staff accept and follow the routine; “*If the patient has a minor complaint the standard may not always be experienced as relevant. In those cases, there may be a failure to comply*” (paper-based-documentation). Individual decisions were described to affect the vital sign data as well as the quality of the care given. In one of the interviews, this type of individually based triage was described “*the most dangerous of triage practices*” (digital documentation). However, some of the interviews mentioned that this practice may be more common among experienced staff. If a conscious decision to diverge from the standard was made by experienced staff members, this was perceived as having less impact on the quality of care. The effect on the vital sign data quality would be the same regardless of staff experience.

A well-defined workflow was experienced to increase the number of complete vital sign measurements. The triage process was an example used in all interviews. However, to be fit for use in CDSS the vital signs also had to be registered in the EHR, as is further discussed under documentation support.

##### Management

Management factors were found to impact the data quality of the vital signs. Opportunities for a quality increase were seen by controlling the quality and giving feedback on performance, but also by leading the organization through resistance to change. When discussing quality management both local and government control were mentioned as important. "*We have done manual record reviews to check the documentation of vital signs. We do regular sample checks of vital sign registrations and give feedback to the staff*" (mixed documentation). "*We received feedback from the National Board of Health and Welfare considering our documentation of vital signs. That has made us change our routines on documentation and the way we follow up compliance to the documentation standards*" (digital documentation). The observations supported that feedback on quality was given, for example, quality indicators were found posted on boards in staff areas. Analysed documents showed that feedback focused on comparing results of the measured indicators to set goals.

Change management referred to resistance to change in an organization. "*No, we don´t have any data on triage vital signs in the EHR as we keep that record on paper. It is tradition and routine and we are quite stuck with it.*" (paper-based documentation). At some of the sites such resistance had been overcome "*It wasn´t completely easy to achieve (switch to electronic documentation), but nowadays no one would consider moving back to paper*" (digital documentation). These sites stressed the importance of leadership and management in making the change happen.

##### Competence and knowledge

Understanding the medical equipment, measurement methods and being able to critically evaluate the results was perceived to impact the correctness of the documented vital signs. “*Considering temperature measurements there may be problems due to simple error sources, like wax in the ear*” (mixed documentation). If the limits and error sources were not understood and known this was perceived as leading to incorrect registrations of vital signs.

The importance of clinical competence when evaluating the plausibility of vital signs and the knowledge of error sources in measurement methods were mentioned as related to data quality. This was discussed in relation to the correctness of the registered vital signs. “*You cannot always trust the device. You have to make a clinical validity check. If there is a problem, you may have to recheck or change method.*” (digital documentation). The competence and knowledge factors were experienced to be managed by training of the junior staff and by the support given by more experienced colleagues.

#### Information technology

##### Documentation support

The interviews showed that three different documentation practices were in place at the sites (Table 3), and only four of the sites did use a fully digitalised documentation flow of vital signs. All sites had implemented EHRs but these were not perceived as supporting documentation in a mobile fast paced emergency care context where staff and patients were described as mobile but IT systems were experienced to be stationary and not always available to the staff at the point of care. Paper-based records were described as light-weight, portable and easy to use when recording vital signs. “*We use the paper record as an emergency record, we register all vital sign measurements and by that way, they are close at hand without any need to log in to the computer*” (paper-based documentation). When paper-based templates were used and transferred to the EHR the currency of the data might be affected. Even if time stamps were reported to be manually set in the EHR to represent measurement time, this practice was described as inconsistent among staff especially when the workload was high.

Retrospective documentation was also perceived to affect the willingness to reenter data into the EHR. “*It feels like double documentation and double work to transfer the paper documentation into the electronic health record*.” (paper-based documentation). When transferring data to the EHR not all measurements were expected to be registered. At sites where documentation was fully digitalized, according to the interviews, the staff was observed using paper notes to record the values in order to enter them into the computerized record later on. This observational finding contrasted findings in the interviews, where all the documentation workflow was described as done in the EHR.

The use of specific documentation templates for the emergency care process was expected to support correct and complete registrations but none of the sites was observed to provide completeness checks with reminders or other intrusive ways to force completeness of vital sign documentation. The EHRs were observed to give plausibility warnings on values that were out of biological range but for these warnings to work standardized templates had to be used. Automation of documentation was mentioned in the interviews as a possible way of affecting completeness and currency. “*Automatic registration of repeated measurements would really improve documentation. If patients are measured every 15 min, there is no time to manually register all measurements.”* (mixed documentation). No sites had any information exchange in place between of measurement devices and the EHR.

The documentation support was discussed under all aspects of data quality, and mobility and ease of use were found important to support timely and complete registrations of the vital signs. The use of documentation templates for the emergency care process connected the documentation support category to the workflow support and interoperability categories.

##### Workflow support

Entering vital signs into the EHRs was not perceived to support the care process but rather linked with later reuse of information. “*The documentation (in the EHR) may be important as a reference later at the ward*” (mixed documentation). “*In my work at the emergency department the documentation (in the EHR) is not important for the acute care process … I rely on the paper-based triage record*" (mixed documentation). This lack of workflow support was experienced to decrease timely and complete registrations. When discussing expectations on a support that would increase the quality of the registered vital signs, three sub-categories were mentioned. These were overviews of information, reuse of information and mobility in the workflow.

Overviews of information were perceived as important for workflow support. Such overviews included read and write functionality combined with a checklist to support complete registrations of the vital signs. *“(in the EHR) we lack usable functionality that is there in the paper triage record. It provides an overview and a checklist of important information and makes us remember things that are supposed to be done*.” (paper-based documentation). If the information entered in the EHR was reused in the process at the emergency department willingness to enter information was expected to increase. Examples given of such reuse included automatic calculation of triage or warnings scores.

A perceived lack of workflow support in the EHRs was discussed in many of the interviews, but the statements were conflicting, and both at sites using paper and digital documentation there were participants acknowledging good-enough workflow support in the present EHRs. These participants further stated that management was the key factor to fully implement the EHRs. However, the overall impression from the interviews and observations was that the EHRs used at the emergency departments today do not fully support a workflow resulting in current and complete registrations of vital signs.

##### Interoperability

The EHR systems were described as complex with separate modules using different keywords for similar concepts. The lack of terminological binding was described to hinder reuse of information within a system. The staff was reluctant to add multiple entries and preferred to use parts designed for their process. “*We are working in our acute care module. We don’t want to use separate parts of the system making double entries*” (mixed documentation).

Sample documents and observations verified that multiple keywords were used for documentation of vital signs and that keywords lacked both binding terminologies and differed in underlying data types. Data could be entered as free text in one keyword and as a numerical value in another. In some sites using paper-based templates for triage, the template was scanned and stored as an image in the EHR and such information was not reusable in digital systems. These examples showed that even though information exists within a system it may not be available to CDSS.

The interviews discussed that separate systems were used in the emergency care flow. The pre-hospital team used a digitalised system to register vital signs but those registrations could not be retrieved or reused by the hospital EHR. As described in one of the sites using digital documentation; “*Vital signs will be measured in the ambulance and registered in their system. We will manually copy them into our EHR*” (digital documentation). These findings were confirmed by the observations.

As a registration that was not retrievable by the CDSS was perceived as incomplete, the interoperability category was found to be connected to the completeness of registrations. Interviews and observations showed that interoperability of the vital signs was expected to be low, making the vital signs so hard to retrieve that they would be considered unfit for use.

### Fitness for use

The experience of the participants was that the vital signs registered in the EHR would not be fit for use for calculation of warning scores or triage scores. The findings showed that five out of nine sites documented the vital signs on a paper record and that the paper-based documentation was connected to a low completeness of in the EHRs. Although some sites would transfer the registrations to the EHR there would likely be a delay before the vital signs would be available. The time to registration was hard to estimate from the interviews and described as variable from minutes to hours, depending on workload and individual preferences. At sites that were supposed to use a fully digitalized documentation flow, the staff was observed to write down measurements on paper to keep for a later registration in the EHR, and this supported the interview finding that some delay was expected at all sites. The main effect of the documentation practice was perceived to impact completeness and currency of the vital sign registrations in the database. The correctness of the vital signs was perceived to be a minor problem. Although error sources were discussed, these were not expected to lead to frequent incorrect registrations of vital signs.

To be fit for use in CDSS the data has to be shared within and between IT systems and work processes. The concept of Interoperability relates to how ready the vital signs are for generic reuse between IT systems and work processes. It includes the functional view of interoperability according to Kubicek [33] that is technical, syntactic, and semantic and business process interoperability.

The results show that although information does exist within the EHRs it may still be very hard to connect and integrate it into CDSS. Even if the vital signs were correct, complete and current they were still not considered fit for use as they lacked binding to standardized terminology or information models. This was further supported in gathered templates and in the observations, where the findings of multiple keywords, with differing data formats, and lack of terminology binding showed a low semantic interoperability. These findings show that registered vital sign data, in Swedish emergency departments EHRs, are likely to be unfit for use in CDSS due to lack of completeness, currency, and interoperability.

### Opportunities to improve quality

All of the interviews included discussions on how to improve vital sign data quality. Summarising these discussions lead us to a five-step approach for vital sign data quality improvement.

1. Standardize the care process
  Following a standardized process was experienced to be of importance to improve completeness. The triage part of the emergency care process was mentioned to be standardized by all sites, but a standardized re-evaluation of vital signs was generally experienced as a challenge. This was explained by the many different conditions investigated and treated at the emergency department. One of the sites mentioned they had started vital sign rounds where they aimed to recheck and document vital signs every 15–30 min on patients with a triage score indicating high acuity.
2. Improving digital documentation support
  Electronic health records were available but not used for documentation of vital signs at all sites and switching to a digital flow was experienced to improve the completeness of vital signs in the EHR. This switch had been made in four of the sites and although hard to make, the switch was perceived as worthwhile and once made broadly accepted. However, manually registered vital signs in the EHR were not experienced to ascertain timely registrations. Integration of medical devices and transfer of information were discussed in all interviews as possible ways to improve documentation of complete and current vital signs.
3. Provide workflow support
  The findings showed that the EHRs were not perceived to provide the same lightweight, easy to use, workflow support as the paper-based triage records, and further the EHRs were not experienced to deliver a sense of usefulness of the registered vital signs. These factors were found to impact the completeness and currency of the vital signs. To further improve vital sign data quality system developers were encouraged to develop mobile solutions that focus on the support of the emergency department workflow.
4. Ensure interoperability
  The findings show that the EHR systems are not ready to exchange vital sign data. The results showed that different keywords and templates without terminological binding or standardized reference models were used. To ensure that entered vital signs were possible to re-use there was an experienced need for improved interoperability.
5. Perform Quality Control
  Giving feedback on data quality and was experienced as a way to improve completeness. Management focus on data quality was also perceived to serve an educational purpose as discussions on quality and possible error sources were thought to increase staff understanding of the importance of documentation.

## Discussion

This study shows that a number of factors impact the vital sign data quality in the emergency care process. The main themes included factors related to the care process and information technology, and among the care process factors it was considered important to follow a set standardized process to minimise individual variation. Management factors were found important in making the transition to a digitalised documentation flow, but that factors within the information technology theme could facilitate or hinder the use of the provided systems. To facilitate use, the EHRs were encouraged to provide workflow and documentation support directly aiming at the emergency care process. Due to the lack of usability, the overall experience is that the vital signs will not be fit for use in CDSS due to low completeness, currency and lack of interoperability.

Sweden is an early adopter country when it comes to health IT and different types of systems such as laboratory information systems, picture archiving and communication systems and also EHRs were introduced earlier in Sweden than in many other countries. Today the penetration of EHRs in hospital care is 100 % and the number of PC clients is about 1 per employee [25]. In this context, the conditions for achieving high data quality through point-of-care documentation seem to be ideal. However, in our study only four out of nine hospitals had completely digital registration of vital signs. Despite the high coverage, EHR systems do not seem to be fully used in the emergency care setting. Even though EHR systems provide emergency care modules with triage support, there is significant resistance when switching to a fully digital workflow, and this type of barrier to the use of EHRs is well described in other studies [34, 35]. According to our results the low usage is related to usability factors, as the electronic health records in the market today are not perceived to provide the mobile workflow support that is present in the paper-based triage records. We conclude that although the systems provide generic EHR support for health care there seems to be an experienced lack of process and workflow support in emergency care.

Our results show that even with a switch to digital registrations, a paper-based documentation may persist that will cause delays in registration and hinder the reuse in real-time CDSS. This finding, indicating issues with the currency of the data, is in line with other studies. Ward et al. studied the effect of the transition from paper-based emergency care records to EHR and found that there are problems with time stamps of the data [28], and further a study on blood pressure measurement registrations in emergency departments also indicated problems with currency [36]. If the systems used for documentation are not available to the staff at the point of care registrations will be delayed [37, 38]. We would argue that the point of care documentation concept would need to include mobility as patients and staff in a busy emergency department are on the move. Stationary workstations were experienced to hinder timely data collection and limit availability and access to information due to login issues. To stimulate complete and timely vital sign registrations EHRs and CDSS were expected to provide workflow support with a direct sense of usefulness [39, 40]. The results show that calculation support on triage scores and overviews targeting the patient data related to the emergency care process could increase the usability. Without this support the documentation may be regarded as retrospective and the sense of importance may be affected.

The results of this study show that the EHR vital sign data may be complete, correct and current but still very hard to make available and connect to a CDSS. The importance of interoperability to realize the potential of EHRs and CDSS is well described in academic publications and government reports [41, 42]. Furthermore, this study shows that interoperability of the data needs to be discussed when considering data quality, especially when the use case is including reuse of data in CDSS. The use of standardized templates may help increasing semantic interoperability within the EHR [43, 44]. By keeping vital signs registered under specified keywords and consistent data formats the data will be easier to retrieve and connect between systems. Preferably these keywords should be based on reference EHR models and connected to standardized reference terminology and coding systems [45].

Interoperability with medical devices is considered as a way of increasing complete, current and connectable registrations [45, 46]. If medical devices are able to deliver vital signs into the EHR the delay of entry may decrease thereby increasing the currency. The integration of medical devices is also likely to increase completeness as this study shows that repeated measurements of vital signs are not registered in the EHR. It should be emphasised that automatic registrations and decision support must be used in accordance with the clinical practice in order to improve medical quality and safety. If not used with added clinical knowledge and experience, automatic registrations and CDSS may carry a risk of unreflective and not patient-centred practice.

### Limitations

There are a number of limitations to the study. Firstly, as only experienced staff was included this may cause omission of areas specific to inexperienced staff. The transferability of our results is dependent on the sample of participants and by the fact that the results are based on expressed individual experiences. To strengthen the transferability of the results we included a representative sample of both university hospitals and secondary referral centres, using three different EHRs and representing three different work practices. Secondly, the researcher’s knowledge and experience with Swedish emergency departments and CDSS may predispose to conclusions in the analysis. To counter the effects of predispositions within the research group the data analysis has been continuously peer-reviewed. Thirdly, the reliability of the results over time will be affected by the limited time frame of the study and also by the fact that the results are dependent on present workflows and presently used IT systems.

### Implications for clinical practice, EHR/CDSS research and development

Further quantitative research may need to confirm our results regarding vital sign data quality in emergency departments. When studying the vital sign data quality, the aspect of interoperability has to be taken into account. Data cannot be considered fit for use if it is not possible to retrieve and connect between IT systems without significant work on mapping. Completeness may be of special interest in the emergency care context as quality is experienced low regarding repeated measurements. Time stamps and the currency of the data should also be a focus point. The results from this study show that there may be significant quality deficiencies in the currency of the data.

If clinical practice is to benefit from CDSS, it is essential that the documentation flow of vital signs is digitalized. In clinical practice digital templates for vital signs based on standardized reference models with terminology binding need to and can be developed in current IT systems by system administrators and clinical staff. EHR system developers should focus on delivering mobile workflow support within EHRs. If to be implemented in emergency care, CDSS likely need to assure the collection of high-quality vital signs as existing data may not be fit for use.

## Conclusion

This study shows that standardization of the workflow is an important concept when discussing vital sign data quality in Swedish emergency departments. A well-defined workflow including measurement and documentation is experienced to reduce individual variation and increase quality. However, to make sure that the documentation is digitalized, information technology has to provide adequate documentation support, otherwise paper-based documentation will be favoured. Lack of such adequate support was described in all of the interviews. This may be an important factor why only four out of the nine sites used the EHR to document vital signs. Because the EHRs, although present at all sites, were not used to register complete and timely vital signs, the data quality was not perceived to be fit for use in calculation of warning scores. Based on these finding we discuss a five step program to improve vital sign data quality.

## Abbreviations

CDSS, Clinical Decision Support Systems; EHR, Electronic Health Record; MD, Medical doctor; RETTS, Rapid emergency triage and treatment system; RN, Registered nurse; SRC, Secondary referral centre; UH, University hospital.

## Additional file

Additional file 1: Interview guide and observation protocol. The interview guide and observation protocol used in the study. (DOCX 13 kb)
