# Supplementary material for: How to improve vital sign data quality for use in clinical decision support systems? A qualitative study in nine Swedish emergency departments
Source: BMC Med Inform Decis Mak. 2016 Jun 4;16:61. doi: 10.1186/s12911-016-0305-4 (PMC4893236; doi:10.1186/s12911-016-0305-4)
Supplement: Additional file 1: — Interview guide and observation protocol. The interview guide and observation protocol used in the study. (DOCX 13 kb) [file 12911_2016_305_MOESM1_ESM.docx]

**Additional file 1: Interview Guide and observation protocol**

**Interview Guide**

| 1. Consent to participate 2. Please, describe who you are and your experience in emergency care 3. Please, describe the process of measuring vital signs at your department 4. Please, expand on the factors affecting how complete, correct and timely the measurements are. 5. Please, describe the process of vital sign documentation at your department 6. Please, expand on the factors affecting how complete, correct and timely the EHR documentation of the measurements is. 7. If not already covered, what is your experience and opinion on how correct, complete and timely the vital signs are in the EHR database? 8. Please, describe your experience of and opinion about how to improve EHR vital sign data quality. |
| --- |

**Observations reporting guide**

| 1 Observed factors affecting complete, correct and timely measurement of vital signs  2 Observed factors affecting complete, correct and timely documentation of vital signs  3 Collection of EHR templates and paper-based records that are used to document vital signs |
| --- |
